# Deacetylation-mediated interaction of SIRT1-HMGB1 improves survival in a mouse model of endotoxemia

**DOI:** 10.1038/srep15971

**Published:** 2015-11-02

**Authors:** Jung Seok Hwang, Hyuk Soo Choi, Sun Ah Ham, Taesik Yoo, Won Jin Lee, Kyung Shin Paek, Han Geuk Seo

**Affiliations:** 1 From the Department of Animal Biotechnology, Konkuk University, Seoul, Korea; 2 The Department of Nursing, Semyung University, Jechon, Korea

## Abstract

Inflammatory signal-mediated release of high-mobility group box 1 (HMGB1) is a damage-associated molecular pattern or alarmin. The inflammatory functions of HMGB1 have been extensively investigated; however, less is known about the mechanisms controlling HMGB1 release. We show that SIRT1, the human homolog of the *Saccharomyces cerevisiae* protein silent information regulator 2, which is involved in cellular senescence and possibly the response to inflammation, forms a stable complex with HMGB1 in murine macrophage RAW264.7 cells. SIRT1 directly interacted with HMGB1 via its N-terminal lysine residues (28–30), and thereby inhibited HMGB1 release to improve survival in an experimental model of sepsis. By contrast, inflammatory stimuli such as lipopolysaccharide (LPS) and tumor necrosis factor-α promoted HMGB1 release by provoking its dissociation from SIRT1 dependent on acetylation, thereby increasing the association between HMGB1 and chromosome region maintenance 1, leading to HMGB1 translocation. *In vivo* infection with wild-type SIRT1 and HMGB1^K282930R^, a hypo-acetylation mutant, improved survival (85.7%) during endotoxemia more than infection with wild-type SIRT1 and HMGB1-expressing adenovirus, indicating that the acetylation-dependent interaction between HMGB1 and SIRT1 is critical for LPS-induced lethality. Taken together, we propose that SIRT1 forms an anti-inflammatory complex with HMGB1, allowing cells to bypass the response to inflammation.

High-mobility group box 1 (HMGB1), a non-histone chromatin-associated nuclear protein, is an evolutionarily conserved protein that is highly expressed in most eukaryotic cells^1^. Within the nucleus, HMGB1 acts as an architectural protein that can bend DNA and promotes the assembly of nucleoprotein complexes, thereby facilitating numerous nuclear functions including transcription, replication, recombination, repair, and maintenance of genome stability^2^. On the other hand, HMGB1 is released into the extracellular milieu during sterile inflammation and infection^3^. Activated immunocompetent cells, including macrophages^4^,^5^, dendritic cells^6^, and natural killer cells^7^, actively secrete HMGB1 after activation upon exposure to pathogen- or damage-associated molecular patterns including lipopolysaccharide (LPS) and other danger signals. The importance of extracellular HMGB1 signals in disease pathogenesis was established because HMGB1 antagonists and a neutralizing anti-HMGB1 antibody significantly reduce the severity of inflammatory conditions such as sepsis, arthritis, colitis, and ischemia reperfusion^4^,^8^,^9^,^10^. These observations indicate the importance of a mechanistic understanding of HMGB1 release from activated immune cells and the regulatory signaling pathways that control these processes.

Unlike the secretion of most cytokines, HMGB1, which lacks classical secretion signal peptides, is released through endoplasmic reticulum- and Golgi-independent unconventional protein secretion pathways^5^,^11^. HMGB1 has two non-classical nuclear export signals and, therefore, shuttles continually from the nucleus to the cytoplasm; however, the equilibrium is almost completely toward the nuclear accumulation of the protein in quiescent cells^12^. By contrast, HMGB1 translocates from the nucleus to the cytoplasm upon the activation of monocytes by inflammatory signals such as LPS or tumor necrosis factor (TNF)-α through the hyper-acetylation of two major clusters of lysine residues within two nuclear localization sequence (NLS) sites^12^. This acetylation-associated translocation is mediated by chromosome region maintenance 1 (CRM1), a nuclear exportin^13^. Serine phosphorylation by TNF-α is another requisite step for the nucleocytoplasmic translocation of HMGB1 in macrophages^14^. Although these findings suggest that post-translational modifications of HMGB1 are critical for its release, it is unclear how these specific modifications control HMGB1 release^12^,^14^.

SIRT1, a mammalian ortholog of yeast silent information regulator 2, is a NAD^+^-dependent class III protein deacetylase that governs a number of genetic programs acting on a wide range of histone and non-histone substrates^15^,^16^,^17^. SIRT1 emerged as a critical regulator of various metabolic and pathophysiological processes, such as mitochondrial biogenesis, cellular senescence, energy metabolism, stress resistance, and inflammation, by coordinating complex gene expression programs through the deacetylation of histones, transcription factors, and co-regulators^15^,^16^,^17^. In addition, SIRT1 was directly implicated in the modulation of inflammatory responses by deacetylating histones and critical transcription factors such as nuclear factor kappa B and activation protein 1, resulting in the transcriptional repression of various inflammation-related genes^18^,^19^. Furthermore, reduction in the level and activity of SIRT1 is closely correlated with chronic inflammatory conditions^20^. Knockout or knockdown of SIRT1 leads to increased cytokine release, whereas SIRT1 activators inhibit production of TNF-α, monocyte chemoattractant protein 1, and interleukin (IL)-8^21^,^22^, stressing the pivotal role of SIRT1 in cellular inflammatory control and the inflammatory response.

Recently, we and others demonstrated that upregulation and activation of SIRT1 inhibits LPS-primed or caloric restriction-mediated HMGB1 release *in vitro* and *in vivo* by unidentified mechanisms^23^,^24^. Here, we report that HMGB1 release is modulated by SIRT1 in macrophages and an animal model of endotoxemia. SIRT1 physically interacts with and deacetylates HMGB1 at multiple lysine residues located at NLS sites, thereby increasing its association with HMGB1 and leading to retention of HMGB1 in the nucleus. These findings shed light on the regulation of HMGB1 release and have important implications in understanding the molecular mechanism underlying the inflammatory reaction, which may aid and encourage the development of new anti-inflammatory drugs.

## Results

### HMGB1 physically interacts with SIRT1

**Figure 1:**
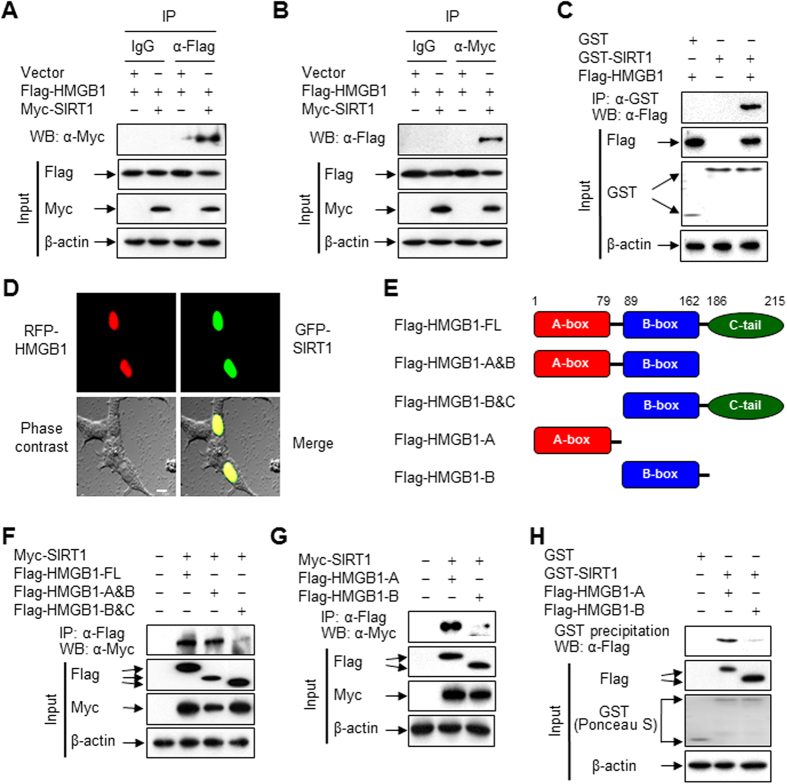
HMGB1 directly interacts with SIRT1. (**A**,**B**) HEK293T cells were co-transfected with the indicated plasmids for 48 h, and then whole-cell lysates were prepared and immunoprecipitated with IgG, anti-Flag, or anti-Myc antibody. The immunoprecipitates and total lysates (input) were subjected to immunoblot analysis with anti-Flag, anti-Myc, and anti-β-actin antibodies to detect HMGB1, SIRT1, and β-actin, respectively. Two percent of whole-cell lysates were used as the input. (**C**) HEK293T cells were transfected with Flag-tagged HMGB1 for 48 h, and whole-cell lysates were incubated with recombinant GST or GST-SIRT1 fusion protein immobilized to glutathione-Sepharose 4B beads for 20 h. Bead-bound proteins were analyzed by Western blotting. GST and GST-fused proteins were stained with Ponceau S. (**D**) The fluorescence of each fusion protein was visualized in HEK293T cells by confocal microscopy. The co-localization of HMGB1 and SIRT1 is indicated by the presence of yellow in the merge image. The bar indicates 10 μm. (**E**) Constructs of Flag-tagged HMGB1 are schematically illustrated. (**F**,**G**) HEK293T cells were co-transfected with Myc-SIRT1 and plasmids harboring Flag-HMGB1 FL or deletion mutants for 48 h. Whole-cell lysates were immunoprecipitated with an anti-Flag antibody. (**H**) HEK293T cells were transfected with Flag-tagged HMGB1 deletion mutants for 48 h. Whole-cell lysates were then prepared and incubated with recombinant GST or GST-SIRT1 fusion protein immobilized to glutathione-Sepharose 4B beads for 20 h. Bead-bound proteins were analyzed by Western blotting. GST and GST-fused proteins were stained with Ponceau S.

Our recent study showed that SIRT1 is a critical factor in the negative regulation of HMGB1 release^23^. To further investigate the detailed mechanism, we examined the interaction between HMGB1 and SIRT1 by co-immunoprecipitation. Lysates of HEK293T cells expressing epitope-tagged proteins were mixed with an anti-Flag antibody, and the resulting immune complexes were analyzed by immunoblotting with an anti-Myc antibody. Immunoprecipitation of HMGB1 from lysates of co-transfected cells resulted in the co-precipitation of SIRT1 (Fig. 1A). We also detected this interaction reciprocally by using an anti-Myc antibody for immunoprecipitation and an anti-Flag antibody for immunoblotting of the precipitate (Fig. 1B). Control mouse immunoglobulin G (IgG) did not precipitate any proteins. Similar conclusions were reached using *in vitro* protein-binding assays in which HMGB1-containing cell lysates were incubated with bacterially produced GST-fused SIRT1 protein (Fig. 1C). Consistent with these results, confocal microscopy showed the co-localization of ectopically expressed red fluorescent protein (RFP)-tagged HMGB1 and green fluorescent protein (GFP)-tagged SIRT1, mainly in the nuclei of HEK293T cells (Fig. 1D).

To identify the regions of HMGB1 that are responsible for its interaction with SIRT1, we generated a panel of HMGB1 deletion mutants (Fig. 1E). These mutants were individually transfected into HEK293T cells together with Myc-SIRT1, and co-immunoprecipitation was performed to evaluate the ability of these mutants to bind to SIRT1. HMGB1-full-length (FL) and HMGB1-A&B displayed a strong interaction with SIRT1, while HMGB1-B&C showed no interaction, suggesting that the N-terminal region of HMGB1 is indispensable for its interaction with SIRT1 (Fig. 1F). To dissect which part of the N-terminal region of HMGB1 interacts with SIRT1, we performed immunoprecipitation with mutants containing only the A-box or B-box. As expected, HMGB1-A interacted strongly with SIRT1, whereas HMGB1-B did not, indicating that the A-box of HMGB1 mediates its interaction with SIRT1 (Fig. 1G). Similar results were obtained by GST pull-down assays in which cell lysates containing HMGB1 deletion mutants were incubated with bacterially produced GST-fused SIRT1 protein (Fig. 1H). These observations provide the first indication that HMGB1 and SIRT1 can form physical complexes with each other.

### LPS promotes the dissociation of HMGB1 and SIRT1 leading to HMGB1 release

**Figure 2:**
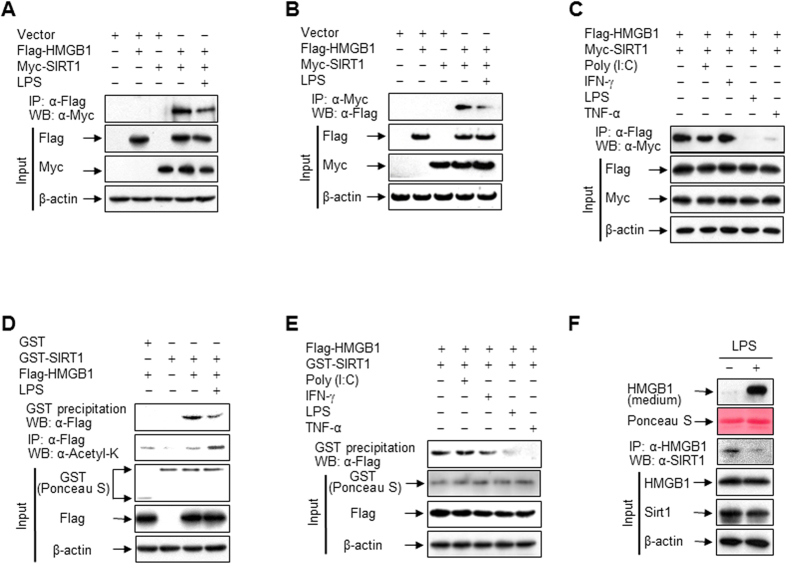
LPS promotes HMGB1 release via its dissociation from SIRT1. (**A–C**) HEK293T cells co-transfected with Flag-HMGB1 and/or Myc-SIRT1 for 48 h were incubated with LPS (100 ng/ml), Poly (I:C) (50 μg/ml), IFN-γ (40 ng/ml), or TNF-α (20 ng/ml) for 3 h, and then whole-cell lysates were immunoprecipitated with an anti-Flag (**A,C**) or anti-Myc (**B**) antibody and analyzed by Western blotting. (**D,E**) HEK293T cells expressing Flag-HMGB1 were incubated with or without the indicated stimuli for 3 h. Whole-cell lysates were incubated with recombinant GST or GST-SIRT1 fusion protein immobilized to glutathione-Sepharose 4B beads for 20 h, and then pulled down or immunoprecipitated. GST and GST-fused proteins were stained with Ponceau S. (**F**) RAW 264.7 cells co-transfected with Flag-HMGB1 and Myc-SIRT1 for 48 h were incubated with or without LPS (100 ng/ml) for 6 h (for interaction) or 24 h (for HMGB1 release). Whole-cell lysates were immunoprecipitated with an anti-HMGB1 antibody to determine the interaction with SIRT1. To detect released HMGB1, equal volumes of conditioned media were analyzed by Western blotting.

HMGB1 released into the extracellular milieu acts as a proinflammatory cytokine in diverse pathological conditions^9^; therefore, homeostatic regulation of this release appears to be essential. We investigated whether SIRT1 functions in this context via its direct interaction with HMGB1. The complex of HMGB1 and SIRT1 dramatically dissociated in the presence of LPS, as judged by co-immunoprecipitations with anti-Flag (Fig. 2A) and anti-Myc (Fig. 2B) antibodies, in HEK293T cells ectopically expressing HMGB1 and SIRT1. Similar results were obtained using another anti-Flag antibody to verify the antibody specificity (Supplemental Fig. S1A). Among stimulators that cause HMGB1 release in monocytic cell lines, including HL-60, U-937, and RAW 264.7 (Supplemental Fig. S1B,C), LPS and TNF-α triggered dissociation of the HMGB1 and SIRT1 complex, while polyinosinic-polycytidylic acid (Poly (I:C)) and interferon (IFN)-γ did not (Fig. 2C). Similar results along with increased acetylation of HMGB1 were observed in a GST pull-down assay using bacterially produced GST-fused SIRT1 protein (Fig. 2D,E). In addition, the complex of HMGB1 and SIRT1 was observed in RAW 264.7 cells without ectopic expression of these proteins under quiescent conditions, and this complex was dissociated in the presence of LPS, leading to the release of HMGB1 into the extracellular milieu (Fig. 2F).

### HMGB1 contains reversibly acetylated lysine residues important for its release

**Figure 3:**
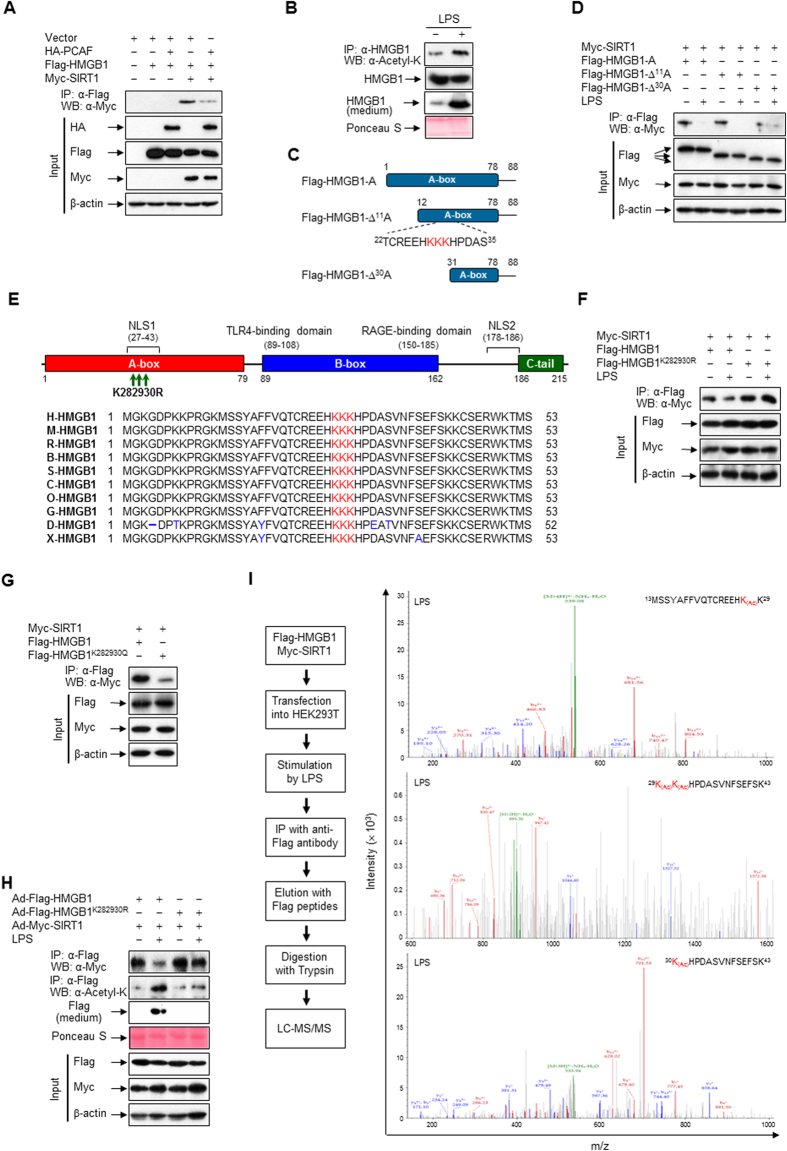
HMGB1 contains reversibly acetylated lysine residues. (**A**) HEK293T cells were co-transfected with HA-PCAF, Flag-HMGB1, and/or Myc-SIRT1 for 48 h, and whole-cell lysates were immunoprecipitated with an anti-Flag antibody and analyzed by Western blotting. (**B**) RAW 264.7 cells were treated with LPS (100 ng/ml) for 6 h (for acetyl-HMGB1) or 24 h (for HMGB1 release), and then whole-cell lysates were immunoprecipitated with an anti-HMGB1 antibody and probed with an anti-acetyl-lysine antibody. Release of HMGB1 was analyzed by immunoblotting the conditioned media. (**C**) Constructs of HMGB1 A-box are schematically shown. (**D**) HEK293T cells co-transfected with Myc-SIRT1 and Flag-HMGB1-A mutants for 48 h were stimulated with LPS (100 ng/ml) for 3 h, and then whole-cell lysates were immunoprecipitated with an anti-Flag antibody. (**E**) Schematic representation of mouse HMGB1 protein with three mutated acetylation residues (lysine 28, 29, and 30) and alignment of the flanking regions of these residues of mouse HMGB1 with those of other species. The conserved lysine residues are highlighted in red. The following abbreviations are used: H, human; M, mouse; R, rat; B, cattle; S, pig; C, dog; O, rabbit; G, chicken; D, zebrafish; X, frog. (**F**,**G**) HEK293T cells co-transfected with Myc-SIRT1 and Flag-HMGB1 or Flag-HMGB1 mutants for 48 h were stimulated with LPS (100 ng/ml) for 3 h, and then whole-cell lysates were immunoprecipitated with an anti-Flag antibody. (**H**) RAW 264.7 cells infected with Ad-HMGB1, Ad-HMGB1^K282930R^, and/or Ad-SIRT1 for 48 h were treated with LPS (100 ng/ml) for 6 h (for acetyl-HMGB1 and protein interaction) or 24 h (for HMGB1 release), and then whole-cell lysates were immunoprecipitated with an anti-Flag antibody. Release of HMGB1 was analyzed by immunoblotting the conditioned media. (**I**) HEK293T cells co-transfected with Myc-SIRT1 and Flag-HMGB1 for 48 h were stimulated with LPS (100 ng/ml) for 3 h, and then whole-cell lysates were immunoprecipitated with an anti-Flag antibody. The immunoprecipitates were digested with trypsin and subjected to liquid chromatography-tandem mass spectrometry (LC-MS/MS) analysis. The fragmentation spectrums of ^13^MSSYAFFVQTCREEHK_(ac)_K^29^, ^29^K_(ac)_K_(ac)_HPDASVNFSEFSK^43^, and ^30^K_(ac)_HPDASVNFSEFSK^43^ revealed the presence of peptides with acetylation at lysine residues 28, 29, and 30, respectively. Procedure for LC-MS/MS analysis is schematically illustrated.

Post-translational modifications such as acetylation are critical for the release of HMGB1 into the extracellular milieu^12^; therefore, we sought to determine whether acetylation affects complex formation between HMGB1 and SIRT1. When monocytic cells were stimulated with diverse signals to release HMGB1, the level of acetylated HMGB1 in immunoprecipitates increased (Supplemental Fig. S1D); the largest increase was over 3-fold in HEK293T cells treated with LPS for 3 h (Supplemental Fig. S1E). To evaluate whether this acetylation of HMGB1 correlated with its dissociation from SIRT1, we used p300/CBP-associated factor (PCAF), which acetylated HMGB1 (Supplemental Fig. S2A). When HA-tagged PCAF was ectopically expressed in HEK293T cells, the association between HMGB1 and SIRT1 was markedly decreased, indicating that PCAF-mediated acetylation of HMGB1 hinders its interaction with SIRT1 (Fig. 3A). This acetylation-mediated dissociation of HMGB1 from SIRT1 was also demonstrated in LPS-stimulated RAW 264.7 cells with increased release of HMGB1 (Fig. 3B). These observations indicate that acetylation of HMGB1 causes it to dissociate from SIRT1, thereby promoting the release of HMGB1 into the extracellular milieu.

The A-box of HMGB1 was a requisite region for its interaction with SIRT1; therefore, we constructed A-box deletion mutants of HMGB1 to identify possible acetylation sites (Fig. 3C). These mutants were individually co-transfected into HEK293T cells together with Myc-SIRT1. A-box and ∆^11^A-box interacted with SIRT1, and this was abolished by LPS, while ∆^30^A-box displayed no interaction with SIRT1, indicating that N-terminal amino acids 12–30 of HMGB1 are essential for its interaction with SIRT1 (Fig. 3D). Similar results were obtained when cells were stimulated with TNF-α (Supplemental Fig. S2B).

To dissect the critical lysine residue(s) responsible for the interaction and dissociation of HMGB1 with and from SIRT1, we analyzed the amino acid sequences within N-terminal 12–30 residues of HMGB1. Within this region, HMGB1 has three lysine residues at positions 28, 29, and 30, which are evolutionarily well-conserved among diverse species (Fig. 3E). To confirm the importance of these lysine residues to the interaction between HMGB1 and SIRT1, we introduced amino acid substitutions at residues 28, 29, and 30 of HMGB1 (HMGB1^K282930R^), replacing the normally present lysine with arginine, which mimics the hypo-acetylation state^25^. When the HMGB1^K282930R^ mutant was co-transfected together with Myc-SIRT1 into HEK293T cells, LPS- or TNF-α-mediated dissociation of HMGB1 and SIRT1 was not observed, in contrast to when wild-type HMGB1 was used (Fig. 3F, Supplemental Fig. S2C). The involvement of acetylation in the dissociation of HMGB1 from SIRT1 was further demonstrated using a mutant in which these three lysine residues were mutated to glutamate (HMGB1^K282930Q^), which mimics the hyper-acetylation state^26^. In this case, complex formation between HMGB1 and SIRT1 was dramatically decreased in quiescent cells in comparison to when wild-type HMGB1 was used (Fig. 3G). However, the individual substitution of any of these three lysine residues did not perturb the dissociation of HMGB1 and SIRT1 in response to LPS or TNF-α, suggesting that acetylation of all three residues underlies this dissociation (Supplemental Fig. S2D,E). These results suggest that lysine residues 28, 29, and 30 of HMGB1 are critical for its interaction with SIRT1 and that inflammatory signal-mediated acetylation of HMGB1 at these lysine residues promotes the relocation of HMGB1 to the cytoplasm, switching a nuclear protein into a cytokine in response to inflammatory stimuli.

To verify that SIRT1 forms a complex with HMGB1 dependent on deacetylation to interfere with the release of HMGB1, adenoviruses expressing wild-type proteins (Ad-Flag-HMGB1 and Ad-Myc-SIRT1) or a deacetylated null mutant of HMGB1 (Ad-Flag-HMGB1^K282930R^) were constructed. Although complexes of HMGB1 and SIRT1 were detected in RAW 264.7 cells co-infected with Ad-Myc-SIRT1 and Ad-Flag-HMGB1 or Ad-Flag-HMGB1^K282930R^, LPS-stimulated dissociation was markedly reduced for the complex of Ad-Myc-SIRT1 and Ad-Flag-HMGB1^K282930R^. This was correlated with inhibition of the release and LPS-induced acetylation of HMGB1 (Fig. 3H). Following infection with these adenoviruses, the levels of exogenous HMGB1 and SIRT1 proteins were similar in the presence and absence of LPS (Fig. 3H, Supplemental Fig. S3A,B). Deacetylation-mediated inhibition of HMGB1 release was confirmed in RAW 264.7 cells treated with LPS or TNF-α (Supplemental Fig. S2F). These results support the hypothesis that HMGB1 and SIRT1 form a complex, maintaining the equilibrium toward the nuclear localization of HMGB1 in quiescent cells.

LPS stimulation rapidly induced the acetylation of HMGB1, which is required for its nuclear translocation and cytoplasmic accumulation^12^; therefore, we determined whether these three lysine resides were acetylated in cells stimulated with LPS or TNF-α. In HEK293T cells transfected with tagged HMGB1 and SIRT1, lysine residues 28, 29, and 30 of HMGB1 were acetylated following stimulation with LPS or TNF-α, as determined by liquid chromatography-mass spectrometry (Fig. 3I, Supplemental Fig. S2G). However, acetylation of all three lysine residues was not detected in HMGB1 isolated from cells stimulated with IFN-γ or Poly (I:C). Although lysine residue 30 of HMGB1 was acetylated in cells stimulated with IFN-γ or Poly (I:C), this is unlikely to be sufficient to stimulate dissociation of the complex of HMGB1 and SIRT1, suggesting that acetylation of all three lysine residues is required for the dissociation of HMGB1 from SIRT1 and its cytoplasmic relocation (Supplemental Fig. S2H, I).

### Nuclear export of HMGB1 via its interaction with CRM1 is negatively regulated by SIRT1

**Figure 4:**
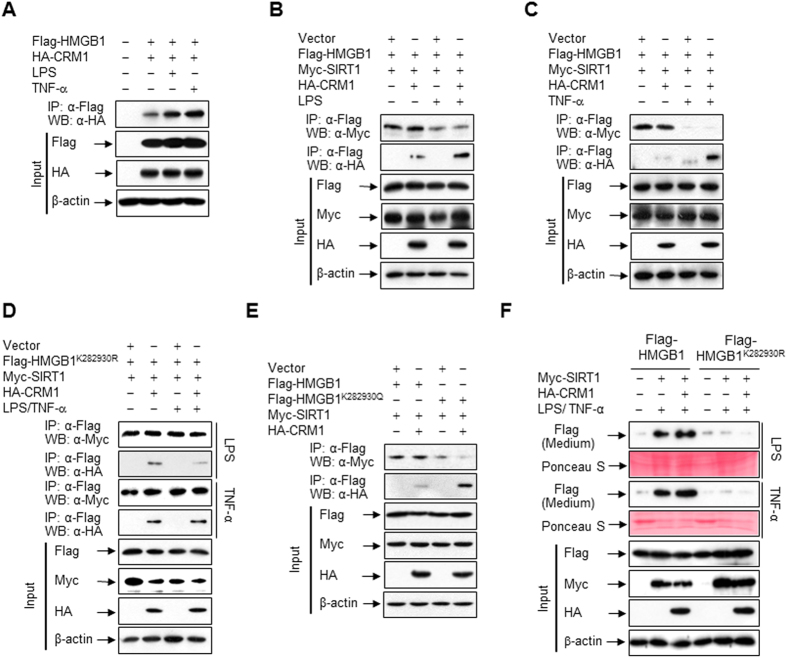
HMGB1 reversibly interacts with CRM1. (**A**–**D**) HEK293T cells co-transfected with Flag-HMGB1, Flag-HMGB1^K282930R^, Myc-SIRT1, and/or HA-CRM1 for 48 h were incubated with LPS (100 ng/ml) or TNF-α (20 ng/ml) for 6 h, and then whole-cell lysates were immunoprecipitated with an anti-Flag antibody and analyzed by Western blotting. (**E**) HEK293T cells were co-transfected with Flag-HMGB1, Flag-HMGB1^K282930Q^, Myc-SIRT1, and/or HA-CRM1 for 48 h, and then whole-cell lysates were immunoprecipitated with an anti-Flag antibody and analyzed. (**F**) RAW 264.7 cells co-transfected with Myc-SIRT1, HA-CRM1, and Flag-HMGB1 or Flag-HMGB1^K282930R^ for 48 h were stimulated with LPS (100 ng/ml) or TNF-α (20 ng/ml) for 24 h. Equal volumes of conditioned media were analyzed by Western blotting to detect released HMGB1.

CRM1 is an evolutionarily conserved protein that is an essential mediator of chromatin structure maintenance and nuclear protein export^27^. To investigate if CRM1 is involved in the export of HMGB1 following its acetylation-mediated dissociation from SIRT1, we examined the interaction between HMGB1 and CRM1 by co-immunoprecipitations. Upon stimulation with LPS or TNF-α, the amount of CRM1 immunoprecipitated with an anti-Flag antibody was increased (Fig. 4A), indicating a potential interaction with HMGB1. By contrast, the interaction between HMGB1 and SIRT1, as judged by co-immunoprecipitations, was substantially attenuated upon stimulation with LPS or TNF-α, suggesting the affinity for HMGB1 is inclined to the CRM1 from SIRT1 (Fig. 4B,C). However, the interaction between CRM1 and HMGB1 was not affected in HEK293T cells transfected with HMGB1^K282930R^, even upon LPS or TNF-α stimulation (Fig. 4D). Furthermore, the interaction between HMGB1 and CRM1 was dramatically increased in HEK293T cells transfected with HMGB1^K282930Q^, even in the absence of stimuli, indicating that acetylation-mediated dissociation of HMGB1 from SIRT1 is critical for the interaction of HMGB1 with CRM1 (Fig. 4E). Next, we examined if this acetylation-mediated interaction of HMGB1 and CRM1 is linked to the release of HMGB1 into the extracellular milieu upon LPS or TNF-α stimulation in RAW 264.7 cells ectopically expressing epitope-tagged proteins. In cells expressing wild-type HMGB1, the extracellular level of HMGB1 was increased upon LPS or TNF-α stimulation, and this was further increased in cells transfected with CRM1, indicating that CRM1 is critical for the shuttling of HMGB1. However, this increased release of HMGB1 was almost completely abolished in cells expressing HMGB1^K282930R^, even in cells transfected with CRM1, suggesting that the deacetylation-mediated interaction between HMGB1 and SIRT1 is important in the regulation of HMGB1 release (Fig. 4F).

### Acetylation is a critical determinant of HMGB1 relocation to the cytoplasm

**Figure 5:**
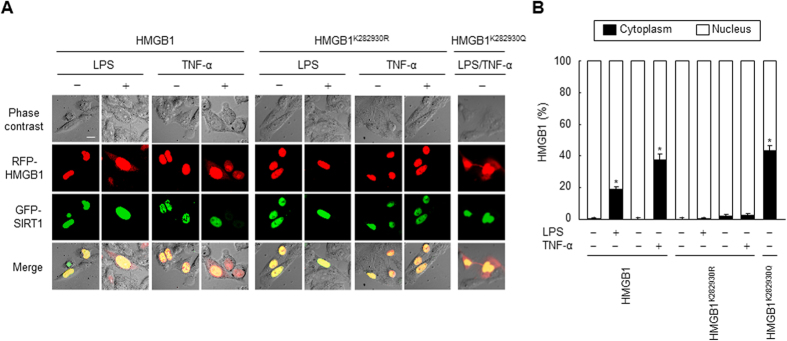
LPS- or TNF-α-induced acetylation of HMGB1 determines its translocation from the nucleus to the cytoplasm. (**A,B**) CHO cells co-transfected with GFP-SIRT1 and RFP-HMGB1, RFP-HMGB1^K282930R^, or RFP-HMGB1^K282930Q^ for 48 h were incubated with or without LPS (100 ng/ml) or TNF-α (20 ng/ml) for 24 h, and then the fluorescence of each fusion protein was visualized by confocal microscopy (**A**) and quantified (**B**). The bar indicates 30 μm. The co-localization of HMGB1 and SIRT1 is indicated by the presence of yellow in the merge images. Results are expressed as the means ± standard error (n = 3). ^*^p < 0.01 compared with the untreated wild-type HMGB1 group.

**Figure 6:**
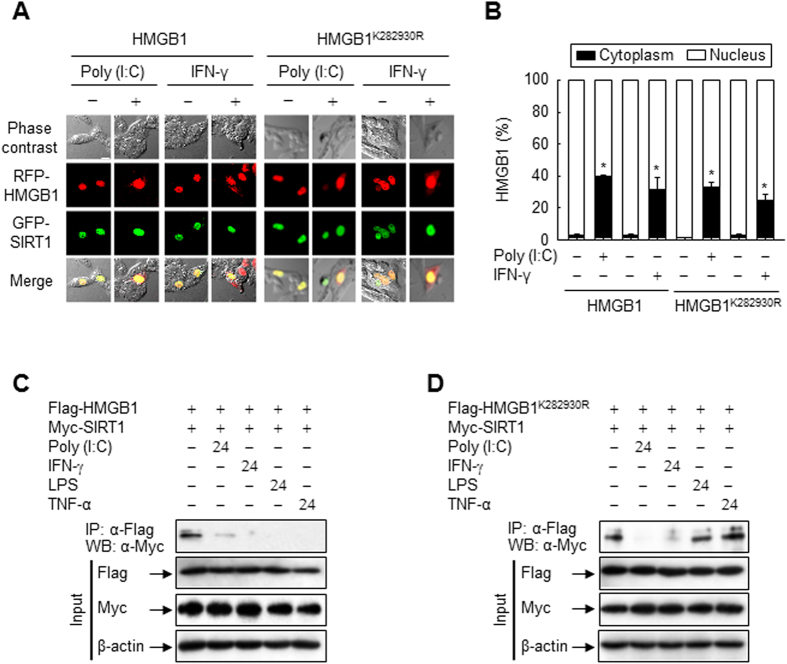
Localizations of HMGB1 and SIRT1 in CHO cells treated with Poly (I:C) or IFN-γ. (**A,B**) CHO cells co-transfected with GFP-SIRT1 and RFP-HMGB1 or RFP-HMGB1^K282930R^ for 48 h were incubated with Poly (I:C) (50 μg/ml) or IFN-γ (40 ng/ml). Following incubation for 24 h, the fluorescence of each fusion protein was visualized by confocal microscopy (**A**) and quantified (**B**). The bar indicates 30 μm. The co-localization of HMGB1 and SIRT1 is indicated by the presence of yellow in the merge images. Results are expressed as the means ± standard error (n = 3). ^*^p < 0.01 compared with the untreated group. (**C,D**) HEK293T cells co-transfected with Myc-SIRT1 and wild-type or mutant Flag-HMGB1 for 48 h were incubated with Poly (I:C) (50 μg/ml), IFN-γ (40 ng/ml), LPS (100 ng/ml), or TNF-α (20 ng/ml) for 24 h, and then whole-cell lysates were prepared and immunoprecipitated with an anti-Flag antibody. The immunoprecipitates and total lysates (input) were subjected to immunoblot analysis with anti-Flag, anti-Myc, and anti-β-actin antibodies to detect HMGB1, SIRT1, and β-actin, respectively. Two percent of whole-cell lysates were used as the input.

To determine the importance of lysine residues 28, 29, and 30 of HMGB1 in its intracellular localization, we further examined the cellular localizations of HMGB1 and SIRT1 using fluorescent fusion proteins of wild-type HMGB1, HMGB1^K282930R^, and SIRT1 by confocal fluorescence microscopy. In Chinese hamster ovary (CHO) cells, wild-type RFP-HMGB1 localized in the nuclear region and co-localized with GFP-SIRT1. Upon stimulation with LPS or TNF-α, although the majority of RFP-HMGB1 protein remained in the nuclear region, numerous signals were detected in the cytoplasm with a diffuse staining pattern. By contrast, this LPS- or TNF-α-induced cytoplasmic localization was almost completely abolished in cells expressing RFP-HMGB1^K282930R^ (Fig. 5A,B). However, this abolishment was not observed in cells stimulated with Poly (I:C) or IFN-γ (Fig. 6A,B). Consistent with these findings, Poly (I:C) and IFN-γ promoted the dissociation of both HMGB1 and HMGB1^K282930R^ from SIRT1, whereas LPS and TNF-α only stimulated the dissociation of the complex between SIRT1 and HMGB1, not between SIRT1 and HMGB1^K282930R^ (Fig. 6C,D). These results indicate that although Poly (I:C) and IFN-γ stimulate HMGB1 release, similar to LPS and TNF-α, in monocytic cells (Supplemental Fig. S1B,C), Poly (I:C)- or IFN-γ-mediated dissociation of HMGB1 from SIRT1 is independent of acetylation of lysine residues 28, 29, and 30 of HMGB1, which is facilitated by LPS and TNF-α.

Hyper-acetylation is a critical signal for the relocation of HMGB1^11^; therefore, we examined a fusion protein with hyper-acetylation mutations (RFP-HMGB1^K282930Q^). The localization of RFP-HMGB1^K282930Q^ was shifted toward the cytoplasm even in the absence of stimuli, similar to the localization of wild-type HMGB1 in the presence of stimuli (Fig. 5A,B). These data suggest that HMGB1^K282930R^ with more nuclear localization is still capable of interacting with SIRT1, while HMGB1^K282930Q^ lose the ability to interact with SIRT1. Therefore, it is most likely that deacetylation is inevitable event for the interaction of HMGB1 and SIRT1. This fits well with the established notion that post-translational modifications of HMGB1, such as acetylation, regulate its release^12^.

### Translocation of HMGB1 is directly regulated by SIRT1

**Figure 7:**
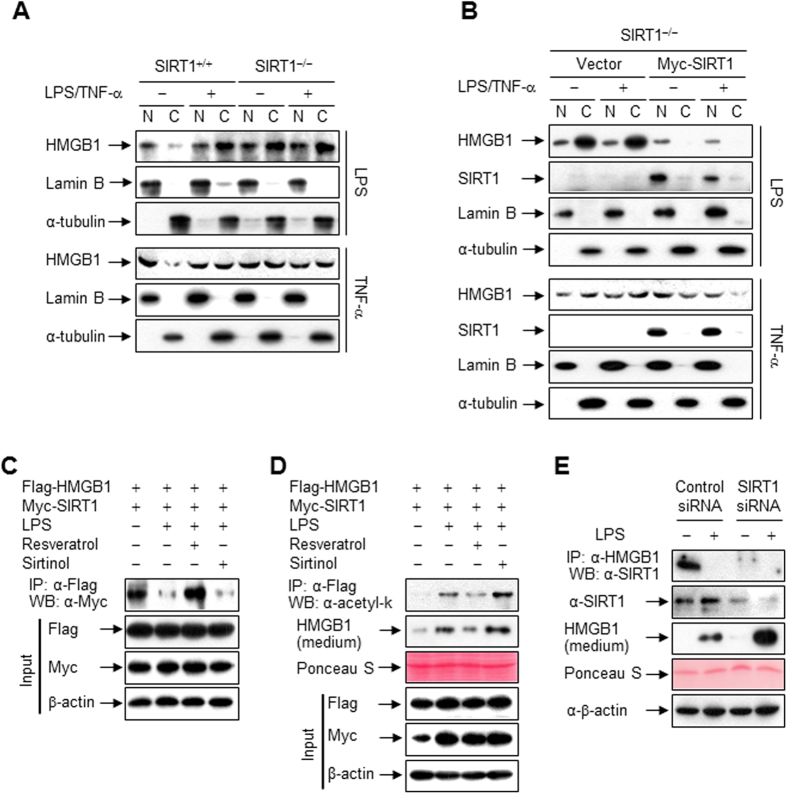
Translocation and release of HMGB1 are directly regulated by SIRT1. (**A**) SIRT1^+/+^ or SIRT1^−/−^ MEFs were treated with LPS (100 ng/ml) or TNF-α (20 ng/ml) for 24 h, and then whole-cell lysates were fractionated into nuclear (N) and cytosolic (**C**) fractions. The localization of HMGB1 was analyzed by Western blotting. (**B**) SIRT1^−/−^ MEFs transfected with an empty vector or Myc-SIRT1 for 48 h were stimulated with LPS (100 ng/ml) or TNF-α (20 ng/ml) for 24 h, and then whole-cell lysates were fractionated. The localizations of HMGB1 and SIRT1 were detected by Western blotting. (**C**) HEK293T cells co-transfected with Flag-HMGB1 and Myc-SIRT1 for 48 h were pretreated with resveratrol (10 μM) or sirtinol (10 μM) for 1 h, and then stimulated with LPS (100 ng/ml) for 3 h. Whole-cell lysates were immunoprecipitated with an anti-Flag antibody and analyzed by Western blotting. (**D**) RAW 264.7 cells co-transfected with Flag-HMGB1 and Myc-SIRT1 for 48 h were pretreated with resveratrol (10 μM) or sirtinol (10 μM) for 1 h, and then stimulated with LPS (100 ng/ml) for 6 h. Whole-cell lysates were immunoprecipitated with an anti-Flag antibody and analyzed. To examine released HMGB1, cells were incubated for 24 h, and then equal volumes of conditioned media were subjected to Western blot analysis. (**E**) RAW 264.7 cells transfected with SIRT1-targeting or control siRNA were stimulated with LPS (100 ng/ml) for 6 h. Whole-cell lysates were immunoprecipitated with an anti-HMGB1 antibody and analyzed. Released HMGB1 was examined as described above.

Mouse embryonic fibroblasts (MEFs) in which *SIRT1* has been genetically deleted (SIRT1^−/−^ MEFs) have significantly increased inflammatory reactions in comparison to wild-type MEFs (SIRT1^+/+^ MEFs)^23^,^28^. Expression of SIRT1 was completely absent in SIRT1^−/−^ MEFs as expected (Supplemental Fig. S4A). When SIRT1^+/+^ MEFs were stimulated with LPS or TNF-α, the translocation of HMGB1 from the nucleus to the cytoplasm was increased, whereas such translocation was observed in SIRT1^−/−^ MEFs regardless of whether the cells were stimulated (Supplemental Fig. S4B). To make a stronger mechanistic connection between SIRT1 and HMGB1 translocation, SIRT1^−/−^ MEFs were transfected with a wild-type SIRT1-expressing vector (Myc-SIRT1). Ectopic expression of SIRT1 prevented translocation of HMGB1 in SIRT1^−/−^ MEFs even in the presence of LPS or TNF-α (Fig. 7B).

To further clarify the functional significance of SIRT1 in HMGB1 release, we assessed the impact of SIRT1 deacetylase activity on the interaction between HMGB1 and SIRT1. Activation of SIRT1 by resveratrol almost completely reversed LPS-induced dissociation of HMGB1 from SIRT1 (Fig. 7C). Regulation of SIRT1 activity by resveratrol or sirtinol, an inhibitor of SIRT1^29^, was also correlated to the acetylation level and release of HMGB1 in RAW 264.7 cells expressing epitope-tagged proteins (Fig. 7D). Furthermore, small interfering RNA (siRNA)-mediated knockdown of SIRT1 reduced the interaction between HMGB1 and SIRT1, thereby increasing the release of HMGB1 from RAW 264.7 cells (Fig. 7E), suggesting that SIRT1 has an anti-inflammatory function by inhibiting HMGB1 release.

### HMGB1 release is correlated with its acetylation status in endotoxemia model mice

**Figure 8:**
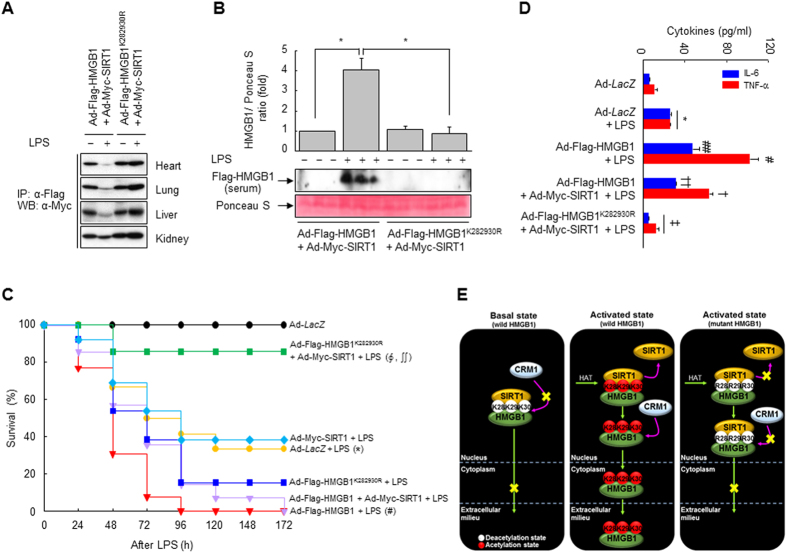
Acetylation-dependent release of HMGB1 via its dissociation from SIRT1 is correlated with endotoxin toxicity. (**A**,**B**,**D**) Tissues were prepared from BALB/c mice infected with Ad-Myc-SIRT1, Ad-Flag-HMGB1, and/or Ad-Flag-HMGB1^K282930R^ at a multiplicity of infection of 0.5 × 10^10^ via the tail vein, followed by the infusion of LPS or vehicle (5 mg/kg, i.p.) 3 days later. Interaction of HMGB1 and SIRT1 (**A**) were analyzed by Western blot and immunoprecipitation using whole-tissue lysates. Circulating levels of Flag-HMGB1 (**B**) and cytokines (**D**) were detected by Western blotting or ELISA, respectively, using sera prepared from samples collected at 16 h post-injection. Results are expressed as means ± standard error (n = 3 or 7). (**C**) BALB/c mice (n = 10–11 per group) were infected with Ad-Myc-SIRT1, Ad-Flag-HMGB1, and/or Ad-Flag-HMGB1^K282930R^ at a multiplicity of infection of 0.5 × 10^10^ via the tail vein, followed by a lethal infusion of endotoxin (LPS, 1 mg/kg, i.p.) 3 days later. Survival was monitored daily for up to 2 weeks. (**E**) Schematic representation of acetylation-dependent interaction of HMGB1 and SIRT1. ^*^p < 0.01 compared with the Ad-*LacZ*-infected group; ^#^*p* < 0.01 and ^##^*p* < 0.05 compared with Ad-*LacZ* + LPS-treated group; ^†^*p* < 0.01 and ^††^*p* < 0.05 compared with the Ad-Flag-HMGB1 + LPS-treated group; ^‡^*p* < 0.01 compared with the Ad-Flag-HMGB1 + Ad-Myc-SIRT1 + LPS-treated group; ^∮^*p* < 0.001 compared with the Ad-Flag-HMGB1 + Ad-Myc-SIRT1 + LPS-treated group; and ^∬^*p* < 0.001 compared with the Ad-Flag-HMGB1^K282930R^ + LPS-treated group.

SIRT1 inhibited LPS- or TNF-α-induced HMGB1 release from macrophages by directly interacting with HMGB1 in an acetylation-dependent manner; therefore, we next analyzed whether SIRT1 affected the circulating HMGB1 level during endotoxemia, a standard model of systemic inflammation. BALB/c mice infected with Ad-Flag-HMGB1, Ad-Flag-HMGB1^K282930R^, and/or Ad-Myc-SIRT1 via the tail vein were challenged with LPS to induce lethal endotoxemia. Expression of Flag-HMGB1, Flag-HMGB1^K282930R^, and Myc-SIRT1 in heart, kidney, liver, and lung was observed in mice infected with adenoviruses in the presence or absence of LPS (Supplemental Fig. S5A,B). Complexes of Flag-HMGB1 and Myc-SIRT1 were detected in co-immunoprecipitated tissue lysates, and this was markedly reduced by LPS treatment. However, LPS-induced suppression of this co-immunoprecipitation was almost completely reversed in the tissues of mice infected with Ad-Flag-HMGB1^K282930R^ and Ad-Myc-SIRT1 (Fig. 8A). In line with these results, the serum level of Flag-HMGB1 was significantly increased in mice infected with Ad-Flag-HMGB1 and Ad-Myc-SIRT1 following endotoxin challenge, whereas it was significantly reduced in mice infected with Ad-Flag-HMGB1^K282930R^ and Ad-Myc-SIRT1 (Fig. 8B). These results support the hypothesis that SIRT1 forms a complex with and deacetylates HMGB1 *in vivo*, thereby inhibiting LPS-induced release of HMGB1.

Particular attention was paid to HMGB1 in the context of LPS-induced endotoxemia, wherein HMGB1 can reportedly exacerbate pathogenic inflammatory responses^4^,^30^. We therefore examined whether the acetylation status of HMGB1 is related to the lethality and survival rate of endotoxemia model mice. When mice were infected with Ad-Flag-HMGB1, their sensitivity to endotoxins was increased (data not shown). Infection of Ad-Flag-HMGB1^K282930R^ and Ad-Myc-SIRT1 conferred significant protection against lethality and improved survival during endotoxemia (survival rate, 85.7%) compared to infection of Ad-Flag-HMGB1^K282930R^ alone (survival rate, 15.3%). This protective effect was not observed in mice infected with Ad-Flag-HMGB1 and/or Ad-Myc-SIRT1, indicating that the acetylation-dependent interaction of HMGB1 and SIRT1 is critical in LPS-induced lethality (Fig. 8C). There were no late deaths of adenovirus-infected animals during the 2 weeks after LPS injection, indicating that SIRT1-mediated inhibition of HMGB1 release conferred lasting protection and did not merely delay the onset of death.

We then examined the serum levels of proinflammatory cytokines that are thought to participate in the pathogenic responses to endotoxemia. The serum levels of TNF-α and IL-6 in mice infected with Ad-Flag-HMGB1 were significantly increased by LPS treatment, while these increases were reduced in the presence of Ad-Myc-SIRT1 (Fig. 8D). Furthermore, infection of Ad-Flag-HMGB1^K282930R^ and Ad-Myc-SIRT1 almost completely abolished LPS-induced secretion of these cytokines, yielding levels similar to those in the control group. These results suggest that SIRT1-mediated hypo-acetylation of HMGB1 attenuates the secretion of proinflammatory cytokines such as TNF-α and IL-6 in endotoxemia, thereby protecting against the LPS-induced clinical manifestations of endotoxemia such as lethargy, diarrhea, and piloerection.

## Discussion

HMGB1, a cytokine as well as a nuclear architectural protein, elicits particular functions depending on its localization^2^,^4^,^8^,^9^,^10^. Although the functions of extracellular HMGB1 in conjunction with its nuclear actions have been well-documented, few of the regulatory mechanisms that determine its cellular localization have been elucidated. In a recent study, we showed that the NAD^+^-dependent deacetylase SIRT1 is an epigenetically regulated anti-inflammatory gene that can functionally cooperate with HMGB1 in cellular inflammation^23^. Here, we present a key mechanism via which post-translational modification of HMGB1 determines its cellular localization, and this process occurs through the interaction of HMGB1 with SIRT1 under inflammatory stimuli. SIRT1 directly interacts with HMGB1, and this protein-protein interaction is favored in quiescent cells. However, this complex dissociates in response to inflammatory signals in an acetylation-dependent manner, leading to the release of HMGB1, a late mediator of endotoxic shock lethality^4^. Loss or gain of SIRT1 function clearly showed that the acetylation level of HMGB1 is intimately related with cellular inflammatory responses^23^,^24^,^28^,^31^. This may indicate a role for the interaction between SIRT1 and HMGB1 in the anti-inflammatory response, i.e., SIRT1-mediated deacetylation inactivates HMGB1 to assist the anti-inflammatory response. In line with this notion, the deacetylation-mediated interaction of HMGB1 and SIRT1 in mice was sufficiently potent to robustly protect against endotoxemia in response to LPS challenge by inhibiting the secretion of HMGB1 and cytokines such as TNF-α and IL-6.

Lysine residues 28, 29, and 30 of HMGB1 were identified as being part of a putative region that mediates the interaction with SIRT1 in an acetylation-dependent manner. Posttranslational modification of HMGB1 reportedly modulates its subcellular localization, either positively or negatively^12^,^32^,^33^. In line with previous studies, inflammatory stimuli induced acetylation of lysine residues 28, 29, and 30 in the N-terminal region of HMGB1, which includes the NLS domain^12^. This stimuli-mediated acetylation promoted the dissociation of HMGB1 and SIRT1, leading to alteration of the subcellular localization of HMGB1. This effect of acetylation on HMGB1 localization correlated with the deacetylase activity of SIRT1, indicating that SIRT1 interacts with and deacetylates HMGB1, thereby preventing its release. Accordingly, acetylation of these sites appears to induce a conformational change in the binding domain of HMGB1 and, hence, alter its interaction with SIRT1. HMGB1^K282930Q^, a hyper-acetylation mutant, exhibited a significantly reduced interaction with SIRT1, while HMGB1^K282930R^, a hypo-acetylation mutant, exhibited an increased interaction with SIRT1 in comparison to wild-type HMGB1, even in the presence of inflammatory stimuli. These findings are consistent with previous studies demonstrating that inflammation- and cellular stress-mediated acetylation of HMGB1 prevents its nuclear reentry and leads to the accumulation of HMGB1 in the cytoplasm^12^,^32^,^33^. Similarly, JAK/STAT- or interferon regulatory factor 1-mediated hyper-acetylation of HMGB1 stimulates its release^11^,^34^. Thus, epigenetic modification of HMGB1 by acetylation has emerged as a critical regulator that can determine the localization of HMGB1. Such findings provide insight into the key role of SIRT1 as a binding partner that maintains HMGB1 in a hypo-acetylated state to inhibit its cytoplasmic accumulation and extracellular release. Accordingly, understanding the mechanisms by which inflammatory cells regulate HMGB1 release may enable the targeting of therapeutics to attenuate HMGB1-related inflammation by the selective activation or expression of the SIRT1

Although HMGB1 is released into the extracellular milieu in response to cellular stimuli such as LPS, TNF-α, IFN-γ, and Poly (I:C), these stimuli did not equally affect the interaction between HMGB1 and SIRT1. In this study, we identified acetylation of lysine residues 28, 29, and 30 of HMGB1 as a key aspect of the regulation of its active release from cells stimulated with inflammatory signals. In line with these findings, LPS and TNF-α induced the acetylation of lysine residues 28, 29, and 30 of HMGB1, whereas IFN-γ and Poly (I:C) only induced the acetylation of lysine residue 30. This difference in the acetylated residues of HMGB1 might be attributed to the induction of different signaling cascades by each stimulus: specifically, LPS transduces signals via Toll-like receptor 4-mediated pathways, while IFN-γ activates JAK-STAT signaling pathways^12^,^35^. Accordingly, it seems most feasible that the location of acetylation determines the signaling cascades that mediate the dissociation of HMGB1 and SIRT1, which, depending on the nature of the stimulus, can lead to the release of HMGB1. During release of HMGB1 following stimulation, HMGB1 is heavily acetylated and relocates to the cytoplasm through an association with CRM1, a nuclear export receptor^5^,^12^. Formation of a complex between HMGB1 and CRM1 accompanies LPS- or TNF-α-induced release of HMGB1^13^,^36^. In addition, leptomycin B, a CRM1 inhibitor, significantly blocks LPS-induced nuclear export of HMGB1^12^. To our knowledge, this is the first study to demonstrate that the specific sites of acetylation modulate HMGB1 release in response to different stimuli via a protein-protein interaction. These novel findings have important implications regarding our understanding of the molecular mechanisms underlying the anti-inflammatory effect of SIRT, as well as the regulation of HMGB1 release.

Of particular interest is the possibility that the acetylation-dependent interaction of SIRT1 and HMGB1 participates in the pathophysiology of sepsis. Pharmacological or genetic manipulation of SIRT1 markedly attenuated LPS- and TNF-α-induced release of HMGB1 in a process mediated by acetylation. Ectopic expression of the hypo-acetylated mutant HMGB1^K282930R^ inhibited LPS-induced increases in the level of circulating HMGB1, indicating that HMGB1 release is tightly regulated by the acetylation status of these residues. Furthermore, expression of HMGB1^K282930R^ reduced endotoxin-induced lethality of LPS in mice. These effects are intimately correlated with the interaction between HMGB1 and SIRT1 as well as the secretion of secondary cytokines such as TNF-α and IL-6 in endotoxemic mouse tissues. These findings are in line with previous studies reporting that HMGB1 is a novel deacetylation target of SIRT1, and that its release and nuclear translocation are intimately linked to SIRT1 deacetylase activity, emphasizing the critical role of SIRT1 in inflammatory responses^23^,^24^,^31^,^37^. In fact, HMGB1 was deacetylated by SIRT1 at four lysine residues (55, 88, 90 and 177) in quiescent endothelial cells^31^. However, these lysine residues were not involved in SIRT1-mediated control of HMGB1 release in the LPS-stimulated murine macrophages. Thus, it may be possible to target SIRT1 to selectively inhibit HMGB1 release without significantly compromising innate immune responses. Modulation of SIRT1 deacetylase activity by pharmacological or genetic manipulation altered the acetylation-dependent release of HMGB1 upon inflammatory stimulation. Consistent with the present findings, pharmacological activation of SIRT1 by resveratrol significantly inhibits HMGB1 release and reduces septic liver injury^24^,^31^,^37^. Accordingly, targeting of SIRT1 in inflammation-related diseases may elicit therapeutic effects by decreasing the extracellular level of HMGB1.

In the current study, we demonstrated that SIRT1 regulates the release of the proinflammatory cytokine HMGB1 via a direct interaction mediated by deacetylation (Fig. 8E). Consequepgntly, the physical interaction between SIRT1 and HMGB1 is associated with a blunted inflammatory response to endotoxin stimuli, leading to a significant increase in the survival of endotoxemic animals.

## Methods

### Materials

Isopropyl-1-thio-β-D-galactopyranoside (IPTG), lipopolysaccharide (LPS, *Escherichia coli* 0111:B4), polyinosinic-polycytidylic acid, Ponceau S, resveratrol, sirtinol, a polyclonal rabbit anti-β-actin antibody, and a monoclonal mouse anti-Flag antibody were obtained from Sigma-Aldrich Co. (St. Louis, MO, USA). Recombinant human polyinosinic-polycytidylic acidinterferon (IFN)-γ and mouse tumor necrosis factor (TNF)-α were obtained from R&D Systems (Minneapolis, MN, USA). Monoclonal rabbit anti-Flag and anti-hemagglutinin (HA) antibodies were obtained from Cell Signaling (Beverly, MA, USA). Monoclonal antibodies specific for acetyl-lysine, α-tubulin, and lamin B, polyclonal antibodies specific for c-Myc and SIRT1, and horseradish peroxidase-conjugated anti-immunoglobulin G were purchased from Santa Cruz Biotechnology (Dallas, TX, USA). A monoclonal rabbit anti-high-mobility group box 1 (HMGB1) antibody was purchased from Epitomics (Burlingame, CA, USA). Other reagents were of the highest grade available.

### Cell culture

RAW 264.7, Chinese hamster ovary, HL-60, U937, and HEK293T cells were obtained from the Korean Cell Line Bank (Seoul, Korea) and maintained in Dulbecco’s modified Eagle’s medium (DMEM) containing 100 U/mL penicillin and 100 μg/mL of streptomycin, supplemented with 10% heat-inactivated fetal bovine serum, at 37 °C under an atmosphere of 95% air and 5% CO_2_. Mouse embryonic fibroblasts derived from wild-type or SIRT1-knockout mice were kindly provided by Dr. Richard Allsopp (Burns School of Medicine, University of Hawaii, Honolulu, HI, USA) and maintained in DMEM as described above.

### Co-immunoprecipitation and immunoblot analysis

Cell or tissue lysates prepared in PRO-PREP Protein Extraction Solution (iNtRON Biotechnology, Seoul, Korea) were pre-cleared with protein G Sepharose™ 4 Fast Flow (GE Healthcare Life Sciences, Buckinghamshire, UK). Pre-cleared lysates were incubated with relevant IgG or the indicated antibodies (1 μg) overnight at 4 °C, and then incubated for 4 h with protein G Sepharose. After washing with phosphate-buffered saline, proteins were extracted from Sepharose beads by boiling in 2× SDS gel-loading buffer and resolved on 10% SDS-polyacrylamide gels. The immunoprecipitates and total lysates (input) were subjected to immunoblot analysis with the indicated antibodies. Immunoreactive bands were detected using West-ZOL Plus (iNtRON Biotechnology). Two percent of whole-cell lysates were used as the input.

### Gene silencing with small interfering RNA (siRNA)

Cells were seeded into 60 mm culture dishes at 18–24 h prior to transfection. siRNA transfection experiments were performed using SuperFect (Qiagen, Valencia, CA, USA) essentially following the manufacturer’s instructions. Transfection-ready control siRNA duplexes were purchased from Ambion (Austin, TX, USA), and SIRT1-targeting siRNA designed against nucleotides (5′-TGA AGT GCC TCA GAT ATT A-3′ and 5′-TAA TAT CTG AGG CAC TTC A-3′) of the mouse *SIRT1* mRNA sequence were synthesized by Bioneer (Daejeon, Korea). Following incubation for 6 h, cells were provided with fresh medium and grown for an additional 38 h, at which point they were treated with the reagents for the indicated period of time. The effects of gene silencing were verified by Western blot analysis.

### Plasmid construction and transfection

HMGB1, p300/CBP-associated factor, and SIRT1 plasmids were constructed as described previously^23^. Deletion mutants of HMGB1 were constructed by PCR amplification of fragments from pcDNA3.1-Flag-HMGB1. These fragments were digested with *Hind*III/*Eco*RV and ligated into the similarly digested epitope-tagged vector pcDNA3.1-Flag (Stratagene, La Jolla, CA, USA), generating pcDNA3.1-Flag-HMGB1-A&B (aa 1–185), pcDNA3.1-Flag-HMGB1-B&C (aa 89–215), pcDNA3.1-Flag-HMGB1-A (aa 1–88), pcDNA3.1-Flag-HMGB1-B (aa 89–185), pcDNA3.1-Flag-HMGB1-Δ^11^A (aa 12–88), and pcDNA3.1-Flag-HMGB1-Δ^30^A (aa 31–88). Site-directed mutants of HMGB1, pcDNA3.1-Flag-HMGB1^K28R^, pcDNA3.1-Flag-HMGB1^K29R^, pcDNA3.1-Flag-HMGB1^K30R^, pcDNA3.1-Flag-HMGB1^K282930R^, and pcDNA3.1-Flag-HMGB1^K282930Q^ were created using a QuikChange Site-Directed Mutagenesis Kit (Stratagene) and the pcDNA3.1-Flag-HMGB1 plasmid. For GST-fused proteins, full-length SIRT1 cDNA was cloned into the *Bam*HI and *Sal*I sites of the pGEX4T-1 vector (GE Healthcare Life Sciences, Pittsburgh, PA, USA). For the localization assay, GFP-SIRT1 and RFP-HMGB1 were generated using pEGFP-C1 and pDsRed-Express-C1 (Clontech Laboratories, Inc., Palo Alto, CA, USA), respectively. The HA-CRM1 plasmid was a gift from Dr. Ralph Kehlenbach (Department of Biochemistry, Georg-August-University of Göttingen, Germany). All recombinant plasmids were verified by sequencing. HEK293T cells transfected with the indicated plasmids for 48 h were stimulated with the indicated reagents for the indicated period of time.

### Fractionation of nuclear and cytoplasmic proteins

Cellular fractions were prepared according to a previously described method^38^. Briefly, cells were washed with PBS, resuspended in lysis solution [10 mM HEPES (pH 7.9), 10 mM KCl, 0.1 mM EDTA, 1 mM DTT, and protease inhibitors], and allowed to swell on ice for 15 min. Nonidet P-40 (0.1%, final concentration) was added, and the sample was vortexed vigorously for 10 sec. The supernatant, a cytosolic fraction, was obtained by centrifugation at 12,000 rpm for 30 sec. The resulting pellet was washed twice with lysis solution and resuspended in PRO-PREP Protein Extraction Solution (iNtRON Biotechnology). After incubation for 30 min on ice, the supernatant fraction containing nuclear proteins was collected by centrifugation at 12,000 rpm at 4 °C for 15 min. The protein concentration was determined by the Bradford method.

### Fluorescence confocal laser microscopy

Cells plated at a density of 1 × 10^5^ cells on coverglass bottom dishes with a diameter of 35 mm were transfected with GFP-SIRT1 and RFP-HMGB1, RFP-HMGB1^K282930R^, or RFP-HMGB1^K282930Q^ using Genefectin (Genetrone Biotech, Gwangmyeong, Korea) essentially following the manufacturer’s instructions. Protein expression was allowed to continue for 48 h after transfection, and cultures were treated with or without LPS or TNF-α for the final 24 h. Following treatment, coverglasses were mounted and observed using an Olympus FV-1000 confocal laser fluorescence microscope (Olympus, Tokyo, Japan).

### Bacterial expression of GST-fused proteins and the GST pull-down assay

Direct binding between SIRT1 and HMGB1 was assessed using bacterially expressed GST-fused proteins as described previously^39^,^40^. Briefly, human SIRT1 cloned into the pGEX4T-1 vector was transformed into BL21 competent cells. A single clone was cultured in LB media containing 50 μg/ml of ampicillin. Expression of GST or GST-fused SIRT1 was induced at an OD_600_ of approximately 0.6 with 0.5 mM IPTG for 18 h at 18 °C. The bacteria pellet was resuspended in lysis buffer [30 mM Tris-Cl (pH 7.5), 0.1 mM NaCl, 1 mM DTT, 1% NP-40, and protease inhibitors], sonicated three times (1 min each time), and centrifuged at 12,000 rpm for 30 min. GST and GST-SIRT1 fusion proteins were largely in the soluble fraction; therefore, supernatants were incubated with glutathione-Sepharose 4B beads (GE Healthcare Life Sciences) for 4 h to immobilize GST or GST-SIRT1. To identify direct binding between SIRT1 and HMGB1, the immobilized GST-fused proteins were incubated with cell lysates overnight at 4 °C. After extensive washing thrice with PBS, the bound proteins were separated by SDS-PAGE and detected by immunoblotting with the indicated antibodies.

### Analysis of HMGB1 release

RAW 264.7 cells were transfected with the indicated plasmids and incubated for 48 h. Following incubation in serum-free medium for 16 h, cells were stimulated with LPS or TNF-α for the indicated period of time. Aliquots of conditioned culture media from equal numbers of RAW 264.7 cells were used to determine the relative amounts of HMGB1 released into the culture media. Equal volumes of conditioned media were precipitated with 80% ice-cold acetone and incubated at −20 °C for 1 h. Protein pellets were precipitated following centrifugation at 13,000 rpm for 10 min at 4 °C. After washing with 80% ice-cold acetone, the pellets were resuspended in SDS-PAGE sample buffer and subjected to Western blot analysis. Ponceau S staining was used to confirm equal loading.

### Viral vector constructs and recombinant viruses

Recombinant adenoviral vectors were constructed for *lacZ*, Flag-HMGB1, Flag-HMGB1^K282930R^, and Myc-SIRT1 expression. All cDNAs were transferred to the pAd/CMV/V5-DEST vector (Invitrogen, Carlsbad, CA, USA) using the Gateway system and LR Clonase II (Invitrogen). The recombinant adenoviral vector was linearized with *Pac*I and transfected into 293A cells using Lipofectamine 2000 (Invitrogen). 293A cells were then cultured for 1–2 weeks in DMEM containing 10% fetal bovine serum. As a control, the pAd/CMV/V5-GW/*lacZ* vector (Invitrogen) was used to produce *lacZ*-bearing adenovirus. All adenoviruses were made in the KIST virus facility (Seoul, Korea).

### Protein purification and mass spectrometry

HEK293T cells were transfected with pcDNA3.1-Flag-HMGB1 and pcDNA3.1-Myc-SIRT1. Following incubation for 48 h, transfected HEK293T cells were treated with LPS or TNF-α for 3 h and then harvested to purify acetylated HMGB1. Cells were collected and lysed in PRO-PREP Protein Extraction Solution (iNtRON Biotechnology), and then whole-cell lysates were prepared and immunoprecipitated with a monoclonal anti-Flag antibody (Sigma-Aldrich). Flag peptide-eluted material was resolved by 10% SDS-PAGE and analyzed as described previously^41^,^42^. The free thiol group of HMGB1 was alkylated for 90 min with 10 mM iodoacetamide at 4 °C. Cysteine residues in disulfide bonds were reduced with 30 mM dithiothreitol at 4 °C for 1 h, followed by alkylation of the newly exposed thiol group with 90 mM *N*-ethylmaleimide at 4 °C for 10 min. Samples were subjected to trypsin digestion according to the manufacturer’s instructions and de-salted using ZipTip C18 pipette tips (Merck Millipore, Billerica, MA, USA). The acetylated lysine residues of HMGB1 were characterized as described previously^43^. All procedures related to mass spectrometry were performed in the Medicinal Bioconvergence Research Center (Suwon, Korea).

### Animal model of endotoxemia and survival test

Endotoxemia was induced in BALB/c mice (male, 6–7-week-old, 20–25 g) by intraperitoneal injection of bacterial endotoxin (10 mg/kg, *Escherichia coli* LPS 0111:B4) as described previously^4^,^44^. Briefly, BALB/c mice were obtained from Koatech (Pyeongtaek, Korea) and housed in a pathogen-free environment. Standard sterilized laboratory diet and water were available *ad libitum* under controlled environmental conditions, with a 12 h light/dark cycle (light on 06:00). Age-matched BALB/c male mice were intravenously injected with 1 × 10^10^ particles of purified recombinant adenoviruses expressing Ad-*LacZ*, Ad-Flag-HMGB1, Ad-Flag- HMGB1^K282930R^, and/or Ad-Myc-SIRT1 in 100 μl of saline. After 2 days, mice were randomly assigned to one of seven groups: injection of vehicle into Ad-*LacZ*-infected mice, injection of LPS (1 mg/kg) into Ad-*LacZ*-infected mice, injection of LPS (1 mg/kg) into Ad-Flag-HMGB1-infected mice, injection of LPS (1 mg/kg) into Ad-Flag-HMGB1 plus Ad-Myc-SIRT1-infected mice, injection of LPS (1 mg/kg) into Ad-Flag-HMGB1^K282930R^-infected mice, injection of LPS (1 mg/kg) into Ad-Flag-HMGB1^K282930R^ plus Ad-Myc-SIRT1-infected mice, and injection of LPS (1 mg/kg) into Ad-Myc-SIRT1-infected mice. Mortality was recorded for up to 2 weeks after LPS injection to ensure that no additional late deaths occurred.

### Ethics statement

All animal studies were performed in accordance to the Korean College of Laboratory Animal Medicine’s guidelines for Animal Care and Use. These regulations were approved by the Institutional Animal Care and Use Committee of Konkuk University (approval number: KU14118).

### Serum cytokine analysis

Serum levels of HMGB1, TNF-α, and interleukin (IL)-6 were analyzed in circulating blood samples obtained from BALB/c mice infected with 1 × 10^10^ particles of purified recombinant adenoviruses expressing Ad-*LacZ*, Ad-Flag-HMGB1, Ad-Flag- HMGB1^K282930R^, and/or Ad-Myc-SIRT1 in 100 μl of saline with or without LPS for 16 h. Blood was collected, allowed to clot for 2 h at room temperature, and centrifuged for 20 min at 4,000 rpm as described previously^45^. Circulating Flag-HMGB1 in serum was determined by Western blot analysis. Serum levels of TNF-α and IL-6 were measured using a mouse TNF-α ELISA Ready-SET-Go! Kit (eBioscience, San Diego, CA, USA) and IL-6 ELISA Max Set Standard (BioLegend, San Diego, CA, USA), respectively.

### Statistical analysis

Data are expressed as means ± standard error. Statistical significance was determined using a one-way ANOVA, followed by the Tukey–Kramer test. A value of *p* < 0.05 was considered statistically significant.

## Additional Information

**How to cite this article**: Hwang, J. S. *et al.* Deacetylation-mediated interaction of SIRT1-HMGB1 improves survival in a mouse model of endotoxemia. *Sci. Rep.* **5**, 15971; doi: 10.1038/srep15971 (2015).

## Supplementary Material

Supplementary Information

## References

[b1] Müller S., Ronfani L. & Bianchi M. E. Regulated expression and subcellular localization of HMGB1, a chromatin protein with a cytokine function. J. Inter. Med. 55, 332–343 (2004). 10.1111/j.1365-2796.2003.01296.x 14871457

[b2] Ueda T. & Yoshida M. HMGB proteins and transcriptional regulation. Biochim Biophys Acta. 1799, 114–118 (2010). 20123073 10.1016/j.bbagrm.2009.11.005

[b3] Andersson U. & Tracey K. J. HMGB1 is a therapeutic target for sterile inflammation and infection. Annu Rev Immunol. 29, 139–162 (2011). 21219181 10.1146/annurev-immunol-030409-101323 PMC4536551

[b4] Wang H. *et al.* HMG-1 as a late mediator of endotoxin lethality in mice. Science 285, 248–251 (1999). 10398600 10.1126/science.285.5425.248

[b5] Gardella S. *et al.* The nuclear protein HMGB1 is secreted by monocytes via a non-classical, vesicle-mediated secretory pathway. EMBO Rep. 3, 995–1001 (2002). 12231511 10.1093/embo-reports/kvf198 PMC1307617

[b6] Dumitriu I. E. *et al.* Release of high mobility group box 1 by dendritic cells controls T cell activation via the receptor for advanced glycation end products. J Immunol. 174, 7506–7515 (2005). 15944249 10.4049/jimmunol.174.12.7506

[b7] Semino C. *et al.* NK/iDC interaction results in IL-18 secretion by DCs at the synaptic cleft followed by NK cell activation and release of the DC maturation factor HMGB1. Blood 106, 609–616 (2005). 15802534 10.1182/blood-2004-10-3906

[b8] Davé S. H. *et al.* Ethyl pyruvate decreases HMGB1 release and ameliorates murine colitis. J Leukoc Biol. 86, 633–643 (2009). 19454652 10.1189/jlb.1008662 PMC2735284

[b9] Harris H. E., Andersson U. & Pisetsky D. S. HMGB1: a multifunctional alarmin driving autoimmune and inflammatory disease. Nat Rev Rheumatol. 8, 195–202 (2012). 22293756 10.1038/nrrheum.2011.222

[b10] Andrassy M. *et al.* High-mobility group box-1 in ischemia-reperfusion injury of the heart. Circulation, 3216–3226 (2008). 18574060 10.1161/CIRCULATIONAHA.108.769331

[b11] Lu B. *et al.* JAK/STAT1 signaling promotes HMGB1 hyperacetylation and nuclear translocation. Proc Natl Acad Sci USA 111, 3068–3073 (2014). 24469805 10.1073/pnas.1316925111 PMC3939889

[b12] Bonaldi T. *et al.* Monocytic cells hyperacetylate chromatin protein HMGB1 to redirect it towards secretion. EMBO J. 22, 5551–5560 (2003). 14532127 10.1093/emboj/cdg516 PMC213771

[b13] Tang D. *et al.* The anti-inflammatory effects of heat shock protein 72 involve inhibition of high-mobility-group box 1 release and proinflammatory function in macrophages. J Immunol. 179, 1236–1244 (2007). 17617616 10.4049/jimmunol.179.2.1236 PMC1976271

[b14] Youn J. H. & Shin J. S. Nucleocytoplasmic shuttling of HMGB1 is regulated by phosphorylation that redirects it toward secretion. J Immunol. 177, 7889–7897 (2006). 17114460 10.4049/jimmunol.177.11.7889

[b15] Bordone L. & Guarente L. Calorie restriction, SIRT1 and metabolism: understanding longevity. Nat Rev Mol Cell Biol. 6, 298–305 (2005). 15768047 10.1038/nrm1616

[b16] Xie J., Zhang X. & Zhang L. Negative regulation of inflammation by SIRT1. Pharmacol Res. 67, 60–67 (2013). 23098819 10.1016/j.phrs.2012.10.010

[b17] Feige J. N. & Auwerx J. Transcriptional targets of sirtuins in the coordination of mammalian physiology. Curr Opin Cell Biol. 20, 303–309 (2008). 18468877 10.1016/j.ceb.2008.03.012 PMC2447870

[b18] Yeung F. *et al.* Modulation of NF-kappaB-dependent transcription and cell survival by the SIRT1 deacetylase. EMBO J. 23, 2369–2380 (2004). 15152190 10.1038/sj.emboj.7600244 PMC423286

[b19] Zhang R. *et al.* SIRT1 suppresses activator protein-1 transcriptional activity and cyclooxygenase-2 expression in macrophages. J Biol Chem. 285, 7097–7110 (2010). 20042607 10.1074/jbc.M109.038604 PMC2844159

[b20] Rajendrasozhan S. *et al.* SIRT1, an antiinflammatory and antiaging protein, is decreased in lungs of patients with chronic obstructive pulmonary disease. Am J Respir Crit Care Med. 177, 861–870 (2008). 18174544 10.1164/rccm.200708-1269OC PMC2292827

[b21] Yang S. R. *et al.* Sirtuin regulates cigarette smoke-induced proinflammatory mediator release via RelA/p65 NF-kappaB in macrophages *in vitro* and in rat lungs *in vivo*: implications for chronic inflammation and aging. Am J Physiol Lung Cell Mol Physiol. 292, L567–576 (2007). 17041012 10.1152/ajplung.00308.2006

[b22] Dong W. *et al.* Inhibitory effects of resveratrol on foam cell formation are mediated through monocyte chemotactic protein-1 and lipid metabolism-related proteins. Int J Mol Med. 33, 1161–1168 (2014). 24584901 10.3892/ijmm.2014.1680

[b23] Hwang J. S. *et al.* Ligand-activated peroxisome proliferator-activated receptor-δ and -γ inhibit lipopolysaccharide-primed release of high mobility group box 1 through upregulation of SIRT1. Cell Death Dis. 5, e1432 (2014). 25275593 10.1038/cddis.2014.406 PMC4649513

[b24] Rickenbacher A. *et al.* Fasting protects liver from ischemic injury through Sirt1-mediated downregulation of circulating Hmgb1 in mice. J Hepatol. 61, 301–308 (2014). 24751831 10.1016/j.jhep.2014.04.010

[b25] Li X. *et al.* SIRT1 deacetylates and positively regulates the nuclear receptor LXR. Mol Cell 28, 91–106 (2007). 17936707 10.1016/j.molcel.2007.07.032

[b26] Schwer B. *et al.* Reversible lysine acetylation controls the activity of the mitochondrial enzyme acetyl-CoA synthetase 2. Proc Natl Acad Sci USA 103, 10224–10229 (2006). 16788062 10.1073/pnas.0603968103 PMC1502439

[b27] Fukuda M. *et al.* CRM1 is responsible for intracellular transport mediated by the nuclear export signal. Nature 390, 308–311 (1997). 9384386 10.1038/36894

[b28] Bernier M. *et al.* Negative regulation of STAT3 protein-mediated cellular respiration by SIRT1 protein. J Biol Chem. 286, 19270–19279 (2011). 21467030 10.1074/jbc.M110.200311 PMC3103305

[b29] Mai A. *et al.* Design, synthesis, and biological evaluation of sirtinol analogues as class III histone/protein deacetylase (Sirtuin) inhibitors. J Med Chem. 48, 7789–7795 (2005). 16302818 10.1021/jm050100l

[b30] El Mezayen R. *et al.* Endogenous signals released from necrotic cells augment inflammatory responses to bacterial endotoxin. Immunol Lett. 111, 36–44 (2007). 17568691 10.1016/j.imlet.2007.04.011 PMC3034364

[b31] Rabadi M. M. *et al.* High-mobility group box 1 is a novel deacetylation target of Sirtuin1. Kidney Int. 87, 95–108 (2014). 24940804 10.1038/ki.2014.217 PMC4270955

[b32] Evankovich J. *et al.* High mobility group box 1 release from hepatocytes during ischemia and reperfusion injury is mediated by decreased histone deacetylase activity. J Biol Chem. 285, 39888–39897 (2010). 20937823 10.1074/jbc.M110.128348 PMC3000970

[b33] Zhang X. *et al.* Calcium/calmodulin-dependent protein kinase (CaMK) IV mediates nucleocytoplasmic shuttling and release of HMGB1 during lipopolysaccharide stimulation of macrophages. J Immunol. 181, 5015–5023 (2008). 18802105 10.4049/jimmunol.181.7.5015 PMC2587501

[b34] Dhupar R. *et al.* Interferon regulatory factor 1 mediates acetylation and release of high mobility group box 1 from hepatocytes during murine liver ischemia-reperfusion injury. Shock 35, 293–301 (2011). 20856174 10.1097/SHK.0b013e3181f6aab0

[b35] Rendon-Mitchell B. *et al.* IFN-gamma induces high mobility group box 1 protein release partly through a TNF-dependent mechanism. J Immunol. 170, 3890–3897 (2003). 12646658 10.4049/jimmunol.170.7.3890

[b36] Hayakawa K., Arai K. & Lo E. H. Role of ERK map kinase and CRM1 in IL-1beta-stimulated release of HMGB1 from cortical astrocytes. Glia 58, 1007–1015 (2010). 20222144 10.1002/glia.20982 PMC3814180

[b37] Xu W. *et al.* Novel role of resveratrol: suppression of high-mobility group protein box 1 nucleocytoplasmic translocation by the upregulation of sirtuin 1 in sepsis-induced liver injury. Shock 42, 440–447 (2014). 25004063 10.1097/SHK.0000000000000225

[b38] Kiemer A. K. *et al.* Phyllanthus amarus has anti-inflammatory potential by inhibition of iNOS, COX-2, and cytokines via the NF-kappaB pathway. J Hepatol. 38, 289–297 (2003). 12586294 10.1016/s0168-8278(02)00417-8

[b39] Davenport A. M., Huber F. M. & Hoelz A. Structural and functional analysis of human SIRT1. J Mol Biol. 426, 526–541 (2014). 24120939 10.1016/j.jmb.2013.10.009 PMC4211926

[b40] Choi K. C. *et al.* Smad6 negatively regulates interleukin 1-receptor-Toll-like receptor signaling through direct interaction with the adaptor Pellino-1. Nat Immunol. 7, 1057–1065 (2006). 16951688 10.1038/ni1383

[b41] Nyström S. *et al.* TLR activation regulates damage-associated molecular pattern isoforms released during pyroptosis. EMBO J. 32, 86–99 (2013). 23222484 10.1038/emboj.2012.328 PMC3545309

[b42] Entezari M. *et al.* Inhibition of extracellular HMGB1 attenuates hyperoxia-induced inflammatory acute lung injury. Redox Biol. 20, 314–322 (2014). 24563849 10.1016/j.redox.2014.01.013 PMC3926109

[b43] Antoine D. J. *et al.* Molecular forms of HMGB1 and keratin-18 as mechanistic biomarkers for mode of cell death and prognosis during clinical acetaminophen hepatotoxicity. J Hepatol. 56, 1070–1079 (2012). 22266604 10.1016/j.jhep.2011.12.019 PMC4127883

[b44] Ulloa L. *et al.* Ethyl pyruvate prevents lethality in mice with established lethal sepsis and systemic inflammation. Proc Natl Acad Sci USA 99, 12351–12356 (2002). 12209006 10.1073/pnas.192222999 PMC129448

[b45] Hwang J. S. *et al.* Activation of peroxisome proliferator-activated receptor γ by rosiglitazone inhibits lipopolysaccharide-induced release of high mobility group box 1. Mediators Inflamm. 2012, 325807 (2012). 10.1155/2012/352807 PMC3539392 23316104

