# Supplementary material for: Deacetylation-mediated interaction of SIRT1-HMGB1 improves survival in a mouse model of endotoxemia
Source: Sci Rep. 2015 Nov 2;5:15971. doi: 10.1038/srep15971 (PMC4629154; doi:10.1038/srep15971)
Supplement: Supplementary Information [file srep15971-s1.pdf]

Supplementary Information

Deacetylation-mediated interaction of SIRT1-HMGB1 improves survival in a mouse model of endotoxemia

Jung Seok Hwang, Hyuk Soo Choi, Sun Ah Ham, Taesik Yoo, Won Jin Lee, Kyung Shin Paek, and Han Geuk Seo

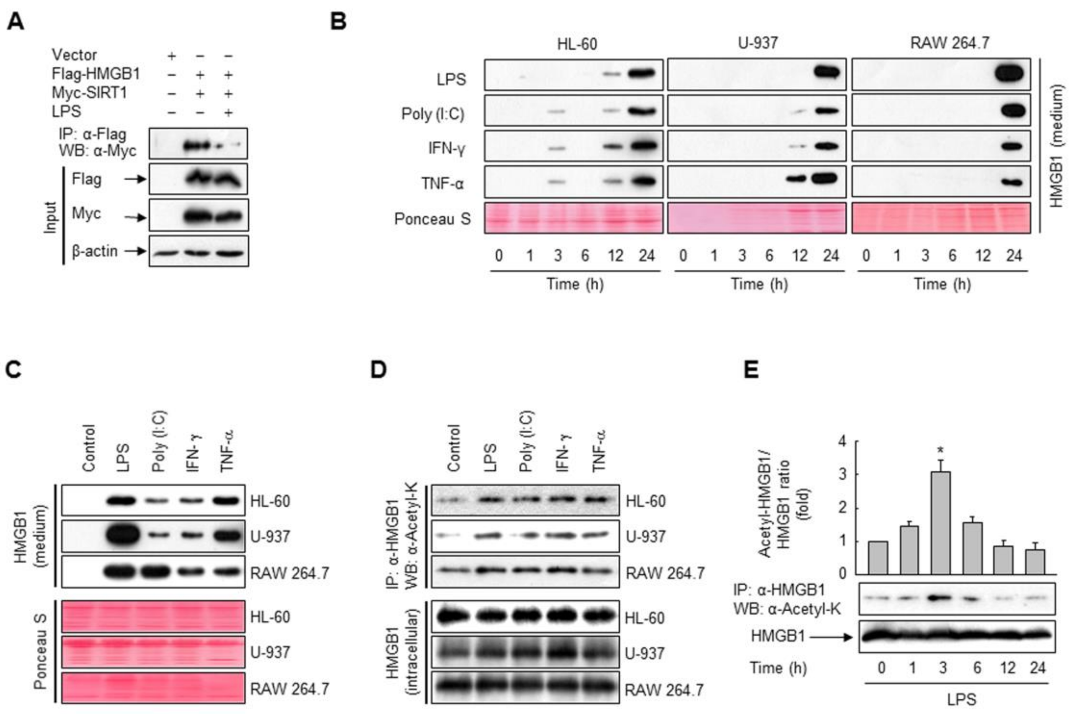

Figure S1. Stimuli-induced release and acetylation level of HMGB1 in monocytic cell lineages. (**A**) HEK293T cells co-transfected with Flag-HMGB1 and Myc-SIRT1 for 48 h were incubated with or without LPS (100 ng/ml) for 3 h, and then whole-cell lysates were prepared and immunoprecipitated with an anti-Flag antibody. The immunoprecipitates and total lysates (input) were subjected to immunoblot analysis with anti-Flag, anti-Myc, and anti-β-actin antibodies to detect HMGB1, SIRT1, and β-actin, respectively. Two percent of whole-cell lysates were used as the input. (**B,C**) Cells seeded in 60 mm culture dishes were allowed to grow to 60% confluency and stimulated with LPS (100 ng/ml), Poly (I:C) (50 μg/ml), IFN-γ (40 ng/ml), or TNF-α (20 ng/ml) for the indicated amount of time (**B**) or 24 h (**C**) in serum-free medium. Equal volumes of conditioned media were subjected to Western blot analysis to detect HMGB1 released into the culture media. Ponceau S staining was used as a loading

control. (**D**) In a parallel group of cells treated with the indicated stimuli for 6 h, whole-cell lysates were prepared and immunoprecipitated with an anti-HMGB1 antibody. The immunoprecipitates were subjected to immunoblot analysis with anti-acetyl-lysine and anti-HMGB1 antibodies to detect acetylated HMGB1 and intracellular HMGB1, respectively. (**E**) HEK293T cells were treated with LPS (100 ng/ml) for the indicated amount of time. Whole-cell lysates were prepared and immunoprecipitated with an anti-HMGB1 antibody, and then subjected to immunoblot analysis with anti-acetyl-lysine and anti-HMGB1 antibodies to detect acetylated HMGB1 and intracellular HMGB1, respectively. Results are expressed as the means ± standard error (n = 3). $^{*}p < 0.01$ compared with untreated group.

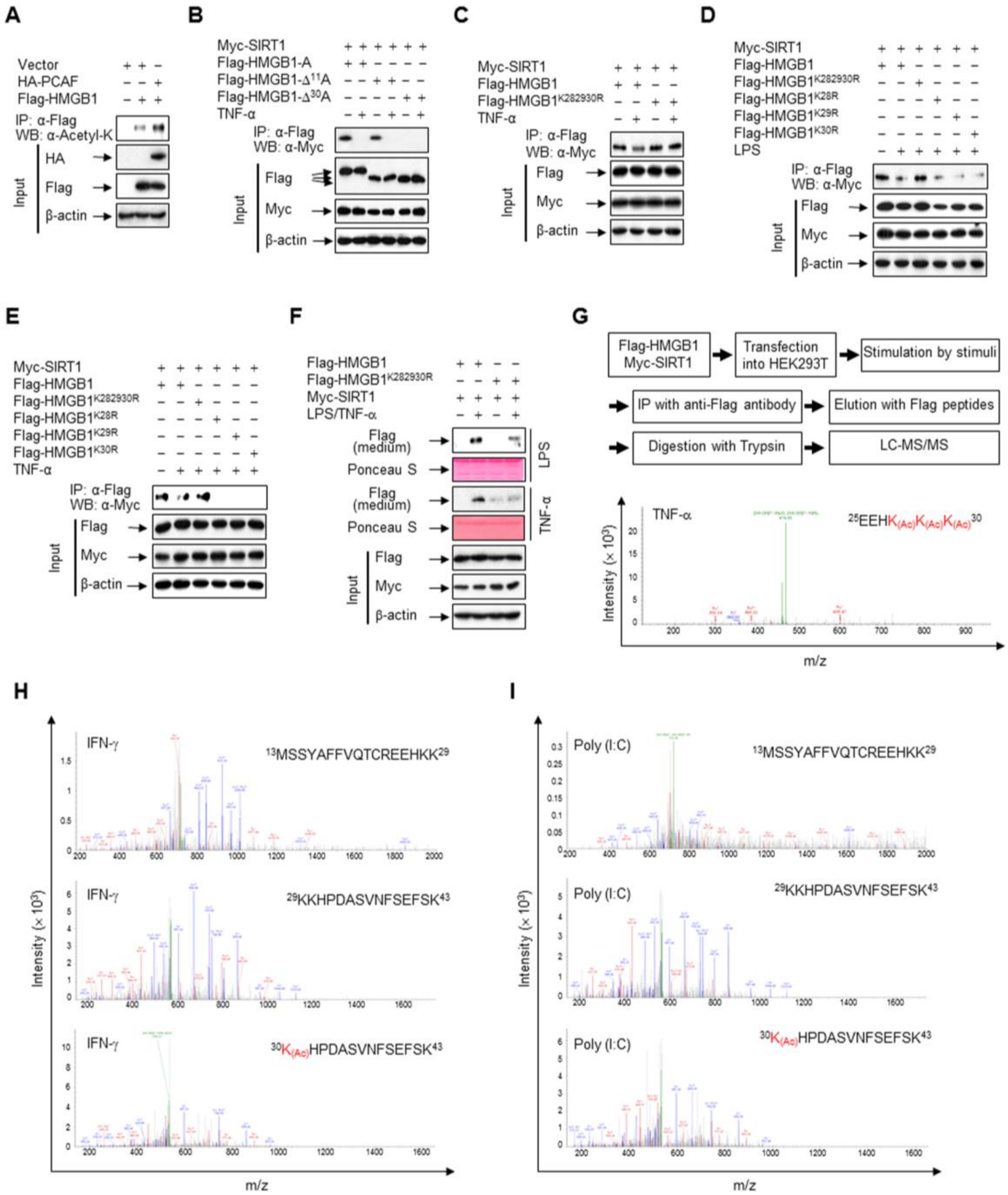

Figure S2. Identification of the domain of HMGB1 responsible for its interaction with SIRT1. (**A**) HEK293T cells were co-transfected with HA-PCAF and Flag-HMGB1 for 48 h, and then whole-cell lysates were prepared and immunoprecipitated with an anti-Flag antibody. The immunoprecipitates and total lysates (input) were subjected to immunoblot analysis with anti-Flag, anti-HA, and anti-β-actin antibodies to

detect HMGB1, PCAF, and β-actin, respectively. Two percent of whole-cell lysates were used as the input. (**B**) HEK293T cells co-transfected with Myc-SIRT1 and Flag-HMGB1 mutants for 48 h were incubated with or without TNF-α (20 ng/ml) for 3 h, and then whole-cell lysates were prepared and immunoprecipitated with an anti-Flag antibody. The immunoprecipitates and total lysates (input) were subjected to immunoblot analysis with anti-Flag, anti-Myc, and anti-β-actin antibodies to detect HMGB1, SIRT1, and β-actin, respectively. (**C-E**) HEK293T cells co-transfected with Myc-SIRT1 and wild-type or mutant Flag-HMGB1 for 48 h were incubated with or without TNF-α (20 ng/ml) or LPS (100 ng/ml) for 3 h, and then whole-cell lysates were prepared and immunoprecipitated with an anti-Flag antibody. The immunoprecipitates and total lysates (input) were subjected to immunoblot analysis as described above. (**F**) RAW 264.7 cells co-transfected with Myc-SIRT1 and Flag-HMGB1 or Flag-HMGB1^{K282930R} for 48 h were incubated with or without TNF-α (20 ng/ml) or LPS (100 ng/ml) for 24 h in serum-free medium. Equal volumes of conditioned media were subjected to Western blot analysis to detect HMGB1 released into the culture media. Ponceau S staining was used as a loading control. (**G**) HEK293T cells co-transfected with Myc-SIRT1 and Flag-HMGB1 for 48 h were incubated with TNF-α (20 ng/ml) for 3 h, and then whole-cell lysates were prepared and immunoprecipitated with an anti-Flag antibody. The immunoprecipitates were digested with trypsin and subjected to LC-MS/MS analysis. The fragmentation spectrum of $^{25}EEHK_{(ac)}K_{(ac)}K_{(ac)}^{30}$ revealed the presence of peptides with acetylation of lysine residues 28, 29, and 30. The protein preparation procedure for LC-MS/MS analysis is schematically illustrated. (**H,I**) HEK293T cells co-transfected with Myc-SIRT1 and Flag-HMGB1 for 48 h were incubated with or without IFN-γ (40 ng/ml) or Poly (I:C) (50 μg/ml) for 3 h, and then

whole-cell lysates were prepared and immunoprecipitated with an anti-Flag antibody. The immunoprecipitates were digested with trypsin and subjected to LC-MS/MS analysis. Acetylation of lysine residue 30, but not of lysine residues 28 or 29, was detected in the fragmentation spectrums of peptides from trypsin-digested HMGB1.

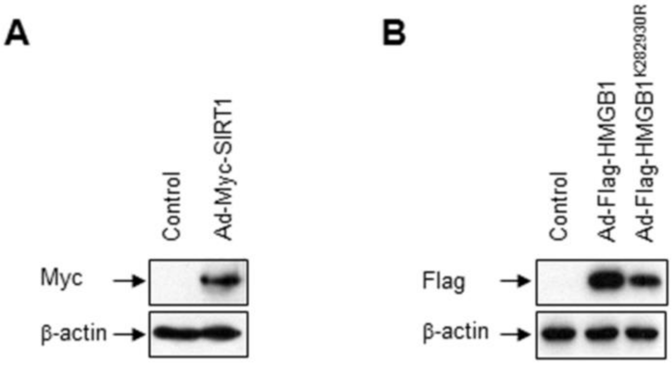

Figure S3. Effects of adenoviral infection on the expression of Ad-Flag-HMGB1, Ad-Flag-HMGB1^{K282930R}, or Ad-Myc-SIRT1. (**A**,**B**) Hela cells were infected with Ad-Flag-HMGB1, Ad-Flag-HMGB1^{K282930R}, or Ad-Myc-SIRT1. Following incubation for 48 h, the cells were lysed, and aliquots of whole-cell lysates were subjected to Western blot analysis with anti-Flag and anti-Myc antibodies to detect adenovirus-mediated expression of Flag-HMGB1 and Myc-SIRT1, respectively.

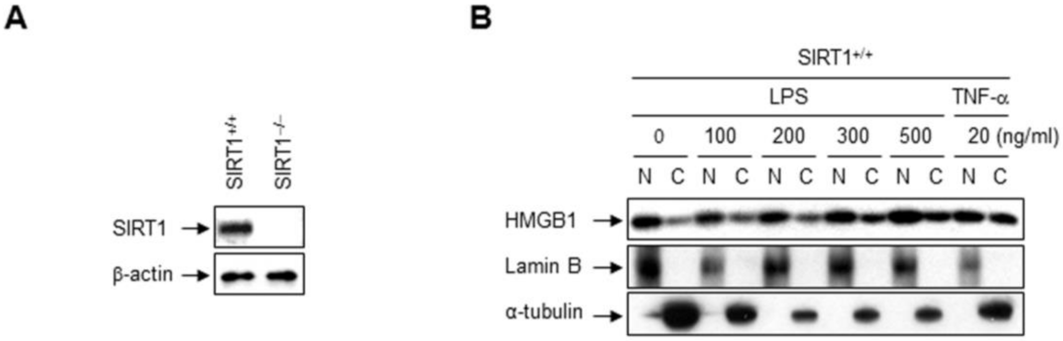

Figure S4. Subcellular localization of HMGB1 in SIRT1$^{+/+}$ and SIRT1$^{-/-}$ MEFs stimulated with LPS or TNF-α. (**A**) Whole-cell lysates were prepared from SIRT1$^{+/+}$ MEFs and SIRT1$^{-/-}$ MEFs, and the expression of SIRT1 was analyzed by Western blotting. (**B**) SIRT1$^{+/+}$ MEFs were treated with the indicated concentrations of LPS or TNF-α for 24 h, and then whole-cell lysates were fractionated into nuclear (N) and cytosolic (C) fractions. The localization of HMGB1 was analyzed by Western blotting with an anti-HMGB1 antibody. Lamin B and α-tubulin were used as loading controls for the nuclear and cytosolic fractions, respectively.

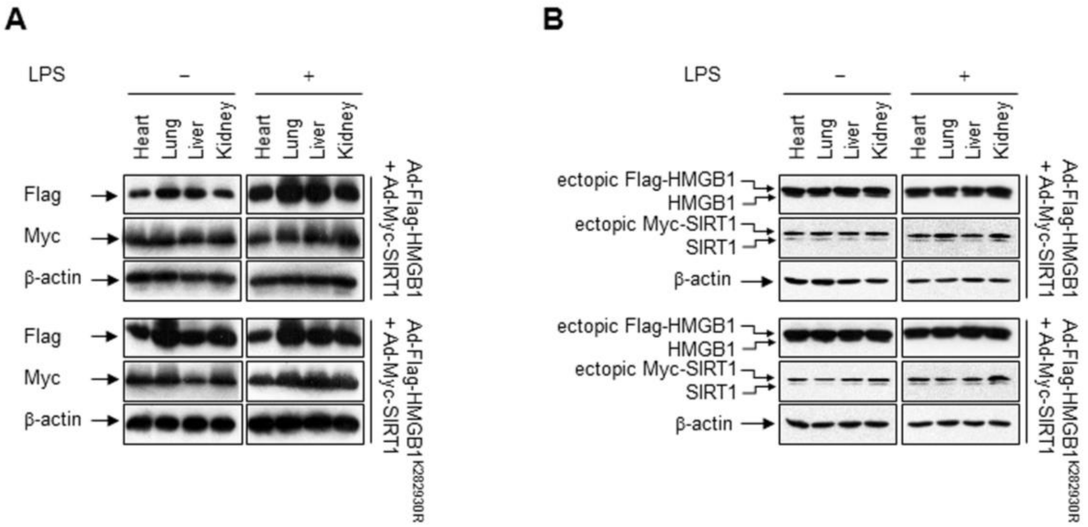

Figure S5. Expression of HMGB1 and SIRT1 in mouse tissues infected with adenoviruses. Tissues were prepared from BALB/c mice infected with Ad-Myc-SIRT1, Ad-Flag-HMGB1, and/or Ad-Flag-HMGB1^{K282930R} at a multiplicity of infection of 0.5×10^{10} via the tail vein, followed by infusion of LPS or vehicle (5 mg/kg, i.p.) 3 days later. Ectopic (A) or endogenous (B) expression levels of HMGB1 and SIRT1 were analyzed by Western blot analysis using whole-tissue lysates.
